# Extreme and moderate temperatures and risk of hospitalizations for pulmonary hypertension: an 11-year time-series study in Shanghai, China

**DOI:** 10.3389/fmed.2026.1771445

**Published:** 2026-03-23

**Authors:** Ang Zhao, Zexi Wu, Xiangyu Yao, Tingting Jiang, Qingxiu Xie, Zhemin Zhang, Zhijing Lin

**Affiliations:** 1 Department of Medical Affair, Shanghai Pulmonary Hospital, Tongji University School of Medicine, Shanghai, China; 2 Department of Toxicology, School of Public Health, Anhui Medical University, Hefei, China; 3 Key Laboratory of Environmental Toxicology of Anhui Higher Education Institutes, Anhui Medical University, Hefei, China; 4 Department of Respiratory Medicine, Shanghai Pulmonary Hospital, Tongji University School of Medicine, Shanghai, China

**Keywords:** hospitalization, pulmonary hypertension, respiratory disease, temperature, time-series analysis

## Abstract

**Background:**

Although ambient temperature is known to influence hospitalizations for respiratory diseases, none has focused specifically on pulmonary hypertension (PH) hospitalizations.

**Methods:**

Using an 11-year dataset (2013–2023) from Shanghai, China, we performed a time-series analysis to assess the impact of daily temperature on PH-related hospitalizations. A distributed lag non-linear model was applied to examine both the immediate (single-day) and prolonged (cumulative) effects of temperature exposure over a lag period of up to 30 days. Cumulative risk estimates were used to assess overall impact of specific temperature exposures by calculating the relative risk (RR) of PH hospitalizations. We also plotted exposure-response curves. Stratification analyses were conducted by age, sex, and medical insurance status to identify potentially vulnerable subpopulations.

**Results:**

Over the study period, a total of 12,218 hospitalizations for PH were recorded. We observed a non-linear, U-shaped relationship between daily mean temperature and PH hospitalization risk. Specifically, cold extremes, including extreme cold (5th percentile of daily average temperature, 4.6 °C) and moderate cold (25th percentile of daily average temperature, 10.4 °C) were significantly linked to increased risk, while no significant associations were observed for heat extremes. Compared with the reference temperature (18 °C), extreme cold showed the strongest effect, with a single-day lag effect peaking at lag 3 (RR: 1.05, 95% CI: 1.01–1.10) and a cumulative effect over lag 0–30 days (RR: 2.80, 95% CI: 1.19–6.59). The attributable fractions of PH hospitalization were 8% for extreme cold and 26% for moderate cold. Stratified analyses revealed higher susceptibility among females, younger individuals (<65 years), and those without medical insurance.

**Conclusion:**

This study provides novel epidemiological evidence that cold exposure significantly increases the risk of PH hospitalization, with particularly pronounced effects observed among females, individuals aged < 65 years, and those without medical insurance.

## Introduction

Pulmonary hypertension (PH), is a progressive and life-threatening cardiopulmonary disorder defined by a average pulmonary arterial pressure ≥ 20 mmHg at rest (1). It is characterized by vascular remodeling, right ventricular (RV) dysfunction, and clinical symptoms such as fatigue and chest pain, ultimately resulting in right ventricular failure and death (2). The etiology of PH involves multifactorial interactions among genetic predisposition, epigenetic dysregulation, metabolic reprogramming, and immune activation, which collectively drive pathological vascular remodeling (3–5). In recent decades, anthropogenic greenhouse gas emissions have accelerated global climate change at an unprecedented rate, making temperature-related health effects a critical priority in environmental epidemiology. Existing evidence has found that temperature adversely affects respiratory health, including mortality (6), hospitalizations (7), and outpatient visits (8). Compared with other organ systems, the respiratory system exhibits heightened sensitivity to temperature fluctuations due to its direct interface with ambient air and its role in thermoregulatory, positioning it as a priority focus in climate-health research. For instance, Zhao et al. identified a reverse J-shaped association between non-optimum temperature and respiratory outpatient visits in northwest China (9), while a systematic review by Turner et al. confirmed elevated respiratory morbidity during heatwaves (10, 11). Besides, multiple studies have linked temperature variability to exacerbated respiratory symptoms (6, 12–14). However, despite extensive research on general respiratory diseases, the effects of temperature on PH, a distinct and severe cardiopulmonary disorder, remain poorly understood. Given the significant clinical and public health burden of PH, investigating its potential sensitivity to temperature extremes represents an important research gap.

Climate change has increased temperature variability, leading to more frequent extreme heat and cold events with significant mortality impacts (15). The Global Burden of Disease (GBD) study reports low temperature as a leading environmental risk factor (16), with 9.4% of global mortality is attributable to non-optimal temperatures (17). While both heat and cold extremes are established risk factors for respiratory mortality, current evidence is limited by broad outcome definitions, methodological inconsistencies, restricted geographic coverage, and unaddressed confounding. Besides, cold-related health burdens substantially surpassed those of heat (18, 19), particularly in warmer regions like Shanghai, where populations show heightened cold vulnerability (20). These patterns underscore the need for disease-specific investigations. PH—a severe cardiopulmonary condition sensitive to environmental stressors—represents a critical research gap. Understanding temperature effects on PH can identify high-risk subgroups (e.g., elderly, uninsured) and inform climate-adaptive health policies, including early warning systems for PH exacerbations during cold spells.

To address the above-mentioned gaps, we performed a time-series study in Shanghai, China, to investigate the impacts of both cold (extreme and moderate) and heat (extreme and moderate) exposures on PH hospitalizations. We further calculated temperature-attributable fractions (AFs) to quantify the population-level burden of excess PH hospitalizations attribute to non-optimal temperatures. We also assessed these patterns among different personal characteristics (age, sex, and medical insurance status) to identify potentially vulnerable subpopulations and to inform targeted public health interventions.

## Materials and methods

### Area

Shanghai (120°52’–122°12’E, 30°40’–31°53’N) is a coastal megacity situated at the eastern terminus of the Yangtze River Delta, bordering the East China Sea. Covering an area of 6,341 square kilometers, the city had a population of 2,487 million at the end of 2023 (21). It exhibits a humid subtropical climate characterized by four distinct seasons and abundant rainfall, with a daily mean temperature reaching 18.5 °C recorded in 2023.

### Health data

Daily data on PH hospitalizations between 1 January 2013 and 31 December 2023 were sourced from the medical record system of a largest three a respiratory hospital in Shanghai, China, which is also one of the largest respiratory and pulmonary medical centers in the region. PH cases were diagnosed based on the International Classification of Diseases (ICD-10) codes: I27.000–I27.202. For each hospitalization, we collected information on patient age, sex, date of hospitalization, and medical insurance status. This study utilized fully anonymized, aggregated hospitalization data; therefore it was exempt from individual informed consent requirements.

### Meteorological data

Daily meteorological data including daily average temperature, relative humidity, atmospheric pressure, and wind speed, were sourced from the Shanghai Meteorological Bureau, a fix-site station located at the center of Shanghai. Daily mean temperature was used as the exposure indicator. Temperature percentiles were defined as follows: the 5th percentile (4.6 °C) represented extreme cold, the 25th percentile (10.4 °C) represented moderate cold, the 75th percentile (25.3 °C) represented moderate hot, and the 95th percentile (31.1 °C) represented extreme hot.

### Statistical analysis

Descriptive statistics, including mean (standard deviation, SD), quartiles (P_25_, P_50_, and P_75_), minimum, and maximum were employed to characterize the demographics of PH hospitalizations and the distribution of meteorological variables between 2013 and 2023.

A quasi-Poisson distributed lag non-linear model (DLNM) was used to evaluate the association between daily mean temperature and PH hospitalizations. This study assumes that the observed daily hospitalization count *Y*_*t*_ follows a distribution proportional to its expected mean *EY*_(t)_. A smooth function of time, represented by a natural cubic spline *ns(time)*, is included in the model to account for autocorrelation in the residuals. It is further assumed that any lagged effects beyond a maximum of 30 days are negligible. The model integrates historical temperature exposure and its lagged structure through a cross-basis matrix, with daily hospitalizations as the outcome variable. The cross-basis matrix was constructed by applying natural cubic splines separately to two dimensions: the exposure-response relationship (daily mean temperature) and the lag-response relationship (lag days 0–30). The tensor product of these two one-dimensional basis functions allows for the simultaneous characterization of non-linear exposure effects and their temporal distribution. The optimal degrees of freedom for both dimensions were selected jointly via a grid search that minimized the quasi-Akaike information criterion (qAIC), thereby balancing model flexibility and parsimony (22). Specifically, we tested df ranging from 3 to 6 for the temperature dimension and 3–5 for the lag dimension. The combination yielding the lowest qAIC (4 df for temperature and 4 df for lag) was selected for the final model. Sensitivity analyses using alternative df specifications confirmed that the main findings were robust to these choices. Relative risks were estimated for specified combinations of temperature and lag, using the mean temperature during the study period as the reference. The model also controlled for potential confounders, including relative humidity, time trends, day of week, holidays, year, atmospheric pressure, and wind speed. The final model specification was as follows:


$$\log\left[E\left(Y_{t}\right)\right]=a+cb\left(Temp_{t,l}\right)+ns\left(time,df=7*year\right)+ns\left(\mathrm{rh},df=4\right)$$

+d⁢o⁢w+h⁢o⁢l⁢i⁢d⁢a⁢y+y⁢e⁢a⁢r+p⁢r⁢e⁢s⁢s⁢u⁢r⁢e+s⁢p⁢e⁢e⁢d

Here, *Y*_*t*_ represents the number of days patients were PH hospitalization, α denotes the intercept, and *cb (Temp_*t,l*_)* represents the cross-basis matrix that combines daily temperature and lag time, where *l* indicates the lag time in days. *ns* refers to the natural cubic spline function, and *df* denotes degrees of freedom. *time* is the time variable, with 7 degrees of freedom each year to control seasonal and temporal trends (23); *dow* and *holiday* are categorical variables to adjust for the effects of weekdays and holidays; *year* represents the year, *pressure* and *speed* represent atmospheric pressure and wind speed, respectively. The reference temperature is the average of the daily average temperatures (T_*mean*_). The cross-basis functions DLNMs were optimized using a quasi-Akaike Information Criterion (qAIC) approach.

To estimate the contribution of temperature to the PH hospital burden, we calculated the AF for both low and high temperatures following established methods (24, 25). The overall AF was computed by aggregating the daily attributable fractions across the entire study period.

Stratified analyses were conducted by age (≥65/<65), sex (female/male), and medical insurance status (yes/no). Differences in effect estimates between subgroups were assessed using a two-sample Z-test (26), where β_1_ and β_2_ are the effect values of two categories (e.g., male/female), and *SE*_1_ and *SE*_2_ are their standard errors. The calculation formula is:


$$z=\frac{\beta_{1}-\beta_{2}}{\sqrt{S\,E_{1}^{2}+S\,E_{2}^{2}}}$$

To test the robustness of our findings, we conducted several sensitivity analyses. First, we examined the influence of model specification by changing the degrees of freedom for the time trend (6, 8, and 9 dfs per year) and for relative humidity (1–7 dfs per year). Second, to formally verify this assumption, we calculated the overdispersion parameter (ϕ) by dividing the Pearson chi-square statistic by the residual degrees of freedom. The estimated ϕ was 1.28, indicated mild overdispersion, confirming that the quasi-Poisson approach was appropriate and that standard Poisson models would have underestimated the standard errors. Finally, we tested the sensitivity of our results to the choice of reference temperature by replacing it with the minimum risk temperature (MRT) as the reference.

Statistical computations were executed by R (version 4.2.3), with “*dlnm*,” “*mgcv*,” “*splines*,” and “*glm*” packages. A two-sided *p*-value of <0.05 was set to determine statistical significance.

## Results

### Descriptive results

The descriptive statistics for daily PH hospitalizations and meteorological variables in Shanghai, 2013−2023 were shown in Table 1. The daily average temperature was 18.2 °C, ranging from −4.6 °C to 35.7 °C. The study included 12,218 hospitalizations for PH, with a daily average of 4.26 admissions. Among these patients, 6,315 (51.7%) were female, 7,125 (58.3%) were aged < 65 years, and 7.439 (60.9%) were uninsured.

**TABLE 1:** Summarized statistics on daily PH hospitalizations and meteorological conditions, 2013–2023.

Variables	Number (%)	Mean (SD)	Min	P_25_	Median	P_75_	Max	IQR
Number of hospitalizations								
Total	12,218 (100.0)	4.3 (2.8)	1	2	4	6	18	4
Sex								
Female	6,315 (51.7)	2.2 (1.8)	0	1	2	3	11	2
Male	5,904 (48.3)	2.1 (1.7)	0	1	2	3	10	2
Age, years								
≥65	5,093 (41.7)	1.8 (1.6)	0	1	1	3	9	2
<65	7,125 (58.3)	2.5 (1.9)	0	1	2	4	11	3
Medical insurance								
Yes	4,779 (39.1)	1.7 (1.5)	0	1	1	2	9	1
No	7,439 (60.9)	2.6 (2.1)	0	1	2	4	14	3
Meteorological conditions								
Mean temp, °C		18.2 (8.6)	−4.6	10.4	18.7	25.3	35.7	14.8
Relative humidity, %		70.9 (14.2)	30	61	72	81	100	20
Pressure, Pa		1016.1 (9.1)	986.4	1008.2	1016.3	1023.3	1042	15.1
Wind speed, m/s		0.6 (0.4)	0	0.3	0.5	0.8	2.9	0.5

PH, pulmonary hypertension; SD, standard deviation; Min, minimum; P_25_, the twenty-fifth percentile; P_75_, the seventy-fifth percentile; Max, maximum; IQR, interquartile range; temp, temperature.

The time-series distributions of daily PH hospitalizations, temperature indexes, and other meteorological variables over the 11-year study period are displayed in Supplementary Figure 1. The results showed no consistent seasonal differences in the association between daily hospitalizations and temperature extremes, supporting the temporal stability of the observed relationships throughout the study period. The correlation among meteorological variables, assessed using Spearman’s rank correlation coefficient, are shown in Supplementary Figure 2.

### Regression results

**FIGURE 1:**
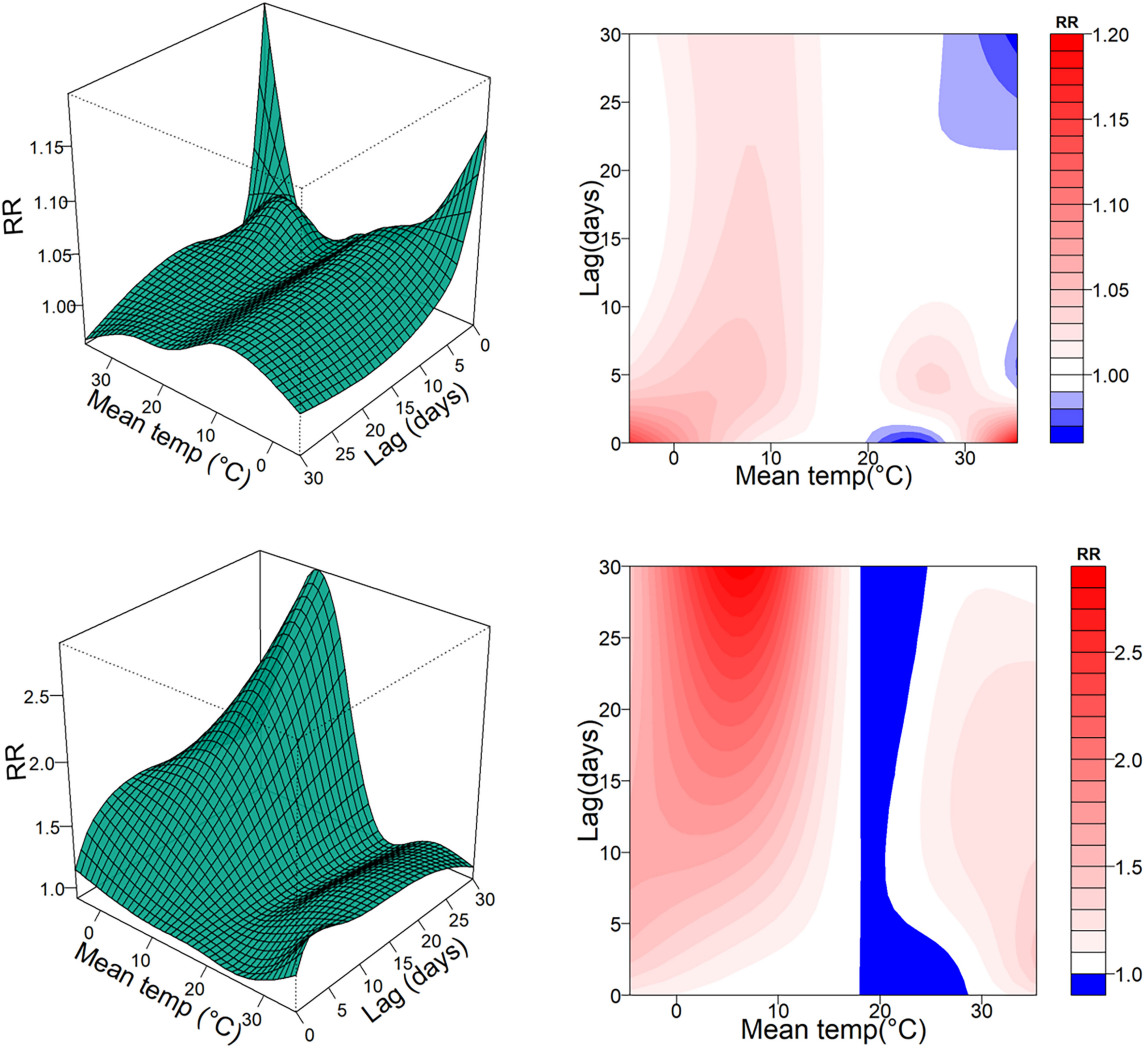
Three-dimensional and contour maps of the single-day and cumulative RRs for PH hospitalizations associated with daily average temperature over lag 0–30 days. PH, pulmonary hypertension; SD, standard deviation; Min, minimum; P_25_, the twenty-fifth percentile; P_75_, the seventy-fifth percentile; Max, maximum; IQR, interquartile range; temp, temperature; RR, relative risk; Mean temp, daily average temperatures.

**FIGURE 2:**
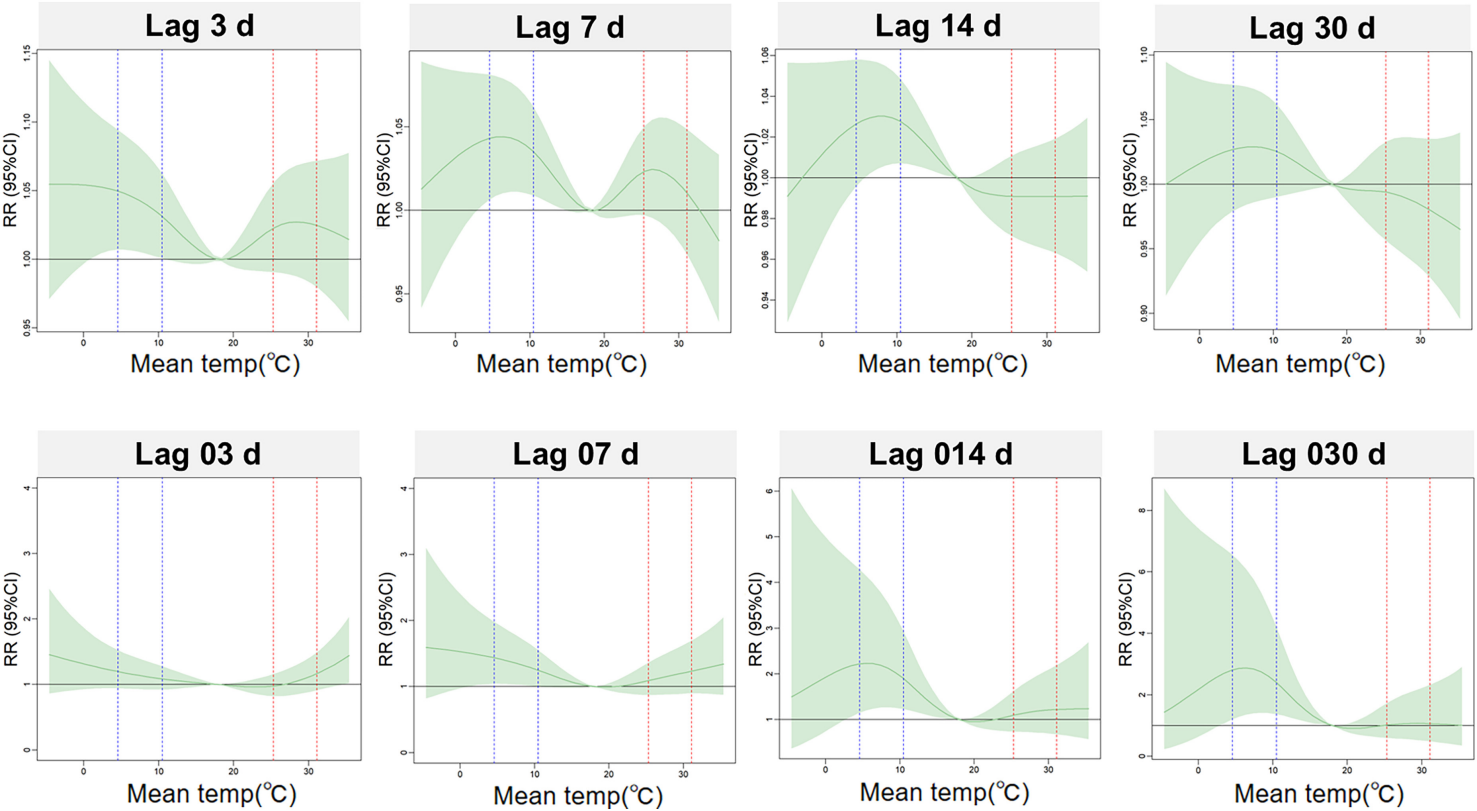
Exposure-response curves for the association between daily average temperature and PH hospitalizations at selected single lags (lag 3, 7, 14, and 30) and cumulative lags (lag 03, 07, 014, and 030). PH, pulmonary hypertension; SD, standard deviation; Min, minimum; P_25_, the twenty-fifth percentile; P_75_, the seventy-fifth percentile; Max, maximum; IQR, interquartile range; temp, temperature; RR, relative risk; Mean temp, daily average temperatures.

Figure 1 shows the three-dimensional exposure-response relationships and corresponding contour plots for the association between daily average temperature and the risk of daily hospitalizations for PH at both single and cumulative lag days. A significantly positive association was observed between temperature and the risk of PH hospitalizations. The overall exposure-response curve was non-linear and non-monotonic. Figure 2 illustrates the exposure-response plots at specific lag periods, using the study-period mean temperature of 18 °C as the reference. Extreme cold (5th, 4.6 °C) and moderate cold (25th, 10.4 °C) were associated with significantly increased overall risks of PH hospitalization. In contrast, the overall effects of moderate hot (75th, 25.3 °C) and extreme hot (95th, 31.1 °C) were not statistically significant.

**FIGURE 3:**
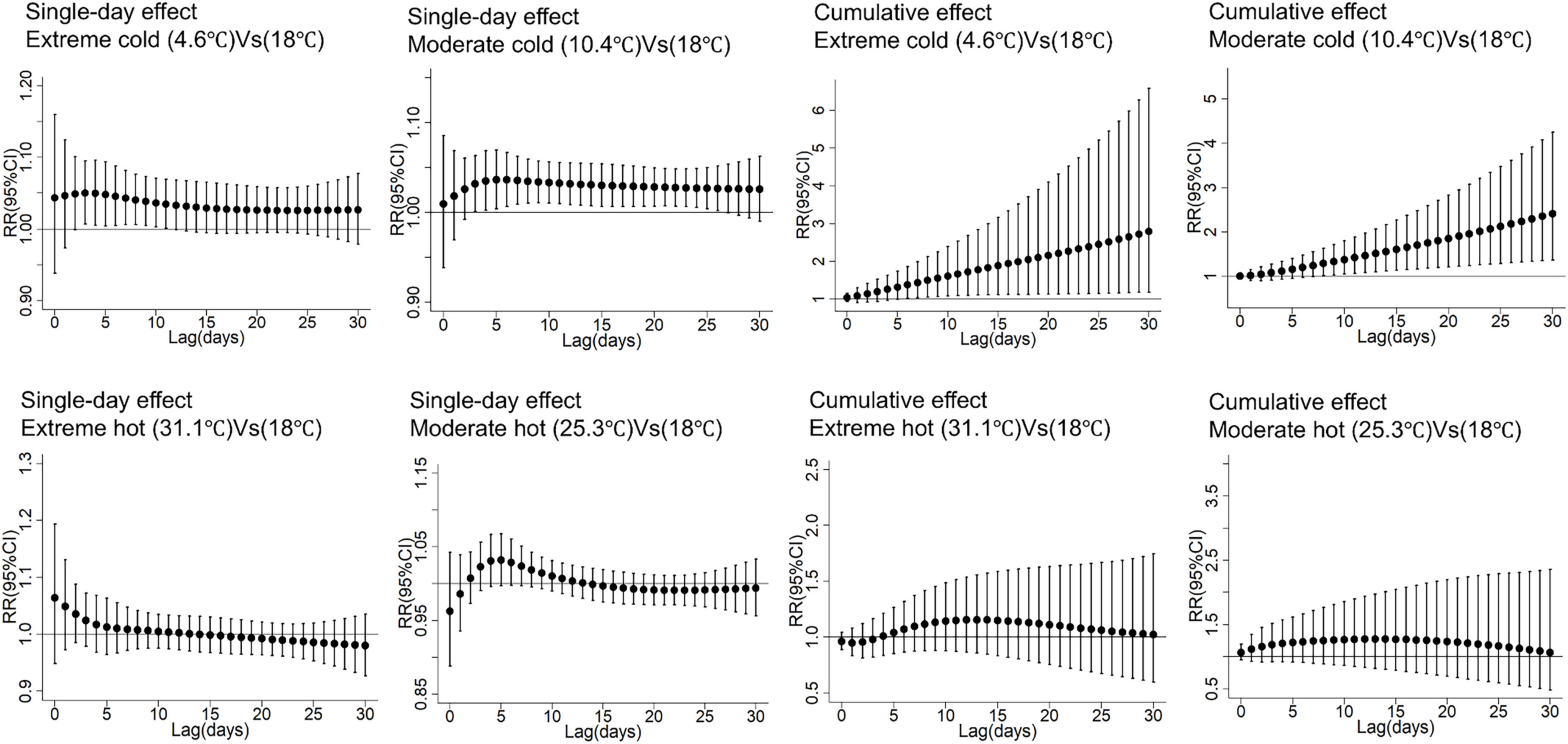
Single and cumulative effects of extreme cold, moderate cold, extreme hot, and moderate hot on PH hospitalizations. Temperature thresholds were defined based on the study-period percentiles of daily mean temperature: extreme cold (5th percentile: 4.6 °C), moderate cold (25th percentile: 10.4 °C), moderate hot (75th percentile: 25.3 °C), and extreme hot (95th percentile: 31.1 °C). The study-period mean temperature (18 °C) served as the reference. PH, pulmonary hypertension; SD, standard deviation; Min, minimum; P_25_, the twenty-fifth percentile; P_75_, the seventy-fifth percentile; Max, maximum; IQR, interquartile range; temp, temperature; RR, relative risk; Mean temp, daily average temperatures.

Figure 3 illustrates the single-day and cumulative lag effects of extreme cold, moderate cold, moderate hot and extreme hot on daily PH hospitalizations. For extreme cold (4.6 °C vs. 18.0 °C), significant single-day effects appeared from lag 3 and persisted through lag 26. For moderate cold, such effects appeared at lag 3 and lasted for 8 days. The greatest single-day effect for extreme cold was observed at lag 3 (RR: 1.05, 95% CI: 1.01–1.10), with the peak effects for moderate cold occurred at lag 5 (RR:1.04, 95% CI: 1.00–1.07).

### Temperature-attributable hospitalization burden

Table 2 presents AFs for PH hospitalizations associated with temperature exposures, stratified by total admissions and different subgroups. Among all temperature categories, moderate cold (25th, 10.4 °C) accounted for the largest proportion of temperature-attributable PH hospitalizations. Subgroup analyses revealed that extreme cold and moderate cold were significantly associated with increased hospitalization risk among females, patients aged < 65 years, and uninsured patients. In contrast, extreme hot significantly contributed to the risk among patients older than 65 years and those with medical insurance.

**TABLE 2:** Attribution fractions for extreme cold, moderate cold, extreme hot, and moderate hot, grouped by sex, age, and medical insurance status.

Variables	Extreme cold	Moderate cold	Extreme hot	Moderate hot
Total	**8% (1%, 22%)**	**26% (9%, 45%)**	1% (−11%, 16%)	0.3% (−3%, 6%)
Sex				
Male	3% (−3%, 17%)	16% (−5%, 42%)	−5% (−22%, 27%)	0% (−3%, 5%)
Female	**16% (3%, 42%)**	**36% (11%, 60%)**	8% (−16%, 43%)	0.1% (−2%, 5%)
Age				
≥65	1% (−3%, 15%)	8% (−9%, 39%)	15% (−14%, 55%)	2% (−2%, 10%)
<65	**17% (3%, 42%)**	**37% (13%, 60%)**	−6% (−22%, 23%)	−0.8% (−3%, 3%)
Medical insurance status				
Yes	4% (−2%, 22%)	6% (−11%, 37%)	31% (−6%, 69%)	**5.6% (0%, 17%)**
No	**13% (1%, 35%)**	**38% (14%, 61%)**	−8% (−23%, 19%)	−1.4% (−3%, 2%)

PH, pulmonary hypertension; SD, standard deviation; Min, minimum; P_25_, the twenty-fifth percentile; P_75_, the seventy-fifth percentile; Max, maximum; IQR, interquartile range; temp, temperature. Bold values denotes statistical significance.

### Stratification analysis

**FIGURE 4:**
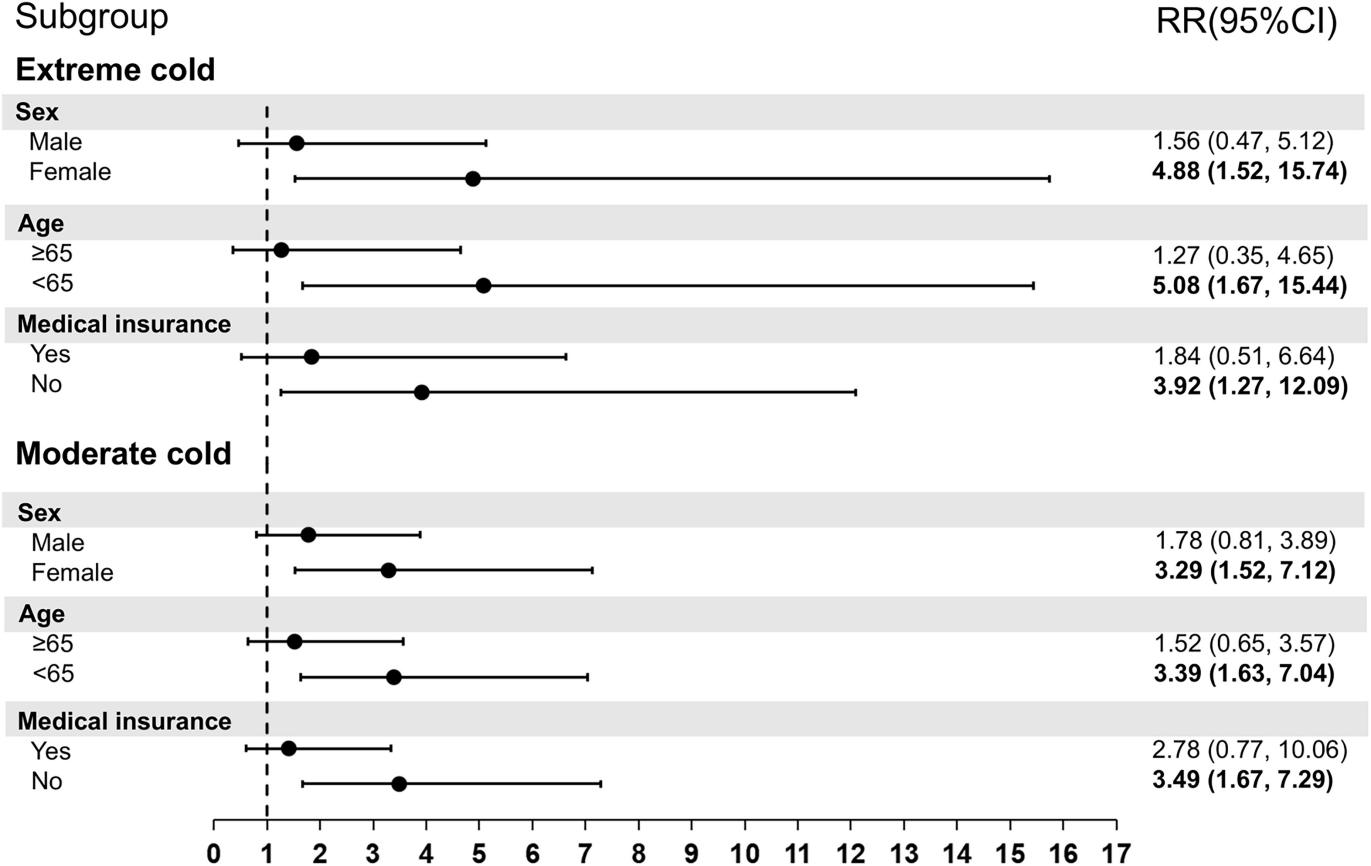
Subgroup-specific relative risks of PH hospitalizations associated with temperature extremes, stratified by sex, age, and insurance status. Bold fonts represent statistical significance (*p*-values < 0.05). PH, pulmonary hypertension; SD, standard deviation; Min, minimum; P_25_, the twenty-fifth percentile; P_75_, the seventy-fifth percentile; Max, maximum; IQR, interquartile range; temp, temperature; RR, relative risk; Mean temp, daily average temperatures.

Figure 4 provides the results of stratified analyses by different demographic factors. Females showed more affected by extreme cold RR:4.88 (95% CI: 1.52–15.74) vs. RR:1.56 (95% CI: 0.47–5.12) and moderate cold RR:3.29 (95% CI: 1.52–7.12) vs. RR:1.78 (95% CI: 0.81–3.89) than males, although the differences were not statistically significant (extreme cold: *Z* = 0.69, *p* = 0.49; moderate cold: *Z* = 0.46, *p* = 0.64). Participants aged < 65 years exhibited greater susceptibility to cold exposure compared with older groups, but this difference did not reach statistical significance. For medical insurance status, uninsured individuals exhibited higher susceptible to cold exposures.

### Sensitivity analysis

Sensitivity analyses assessing the robustness of the relationships of daily average temperature, extreme cold and moderate cold with PH hospitalizations are shown in Supplementary Figures 3, 4. Varying the degrees of freedom for humidity spline (*df* = 1, 2, 4, 5, 6, and 7) did not significantly alter the main results. Similarly, adjusting for the annual degrees of freedom for the time trend (*df* = 8, 9, 10) yielded consistent results, confirming the stability of our primary estimates. Furthermore, replacing the reference temperature with the MRT did not materially alter the results (Supplementary Figure 5).

## Discussion

This study provides novel epidemiological evidence that both extreme cold and moderate cold are significantly associated with an increased risk for PH hospitalization. Although extreme hot showed a relatively strong effect estimate, it did not reach statistical significance, while moderate hot showed no meaningful association. Single-day lag analyses indicated that the risk effects of cold exposure peaked at 3–5 days post-exposure, with cumulative effects increasing progressively over longer lag periods. Notably, we observed substantial heterogeneity in susceptibility to cold exposure across population subgroups. Females, individuals aged < 65 years old, and those without health insurance showed higher susceptibility to clod-related hospitalization. In contrast, although not statistically significant, the risk associated with high temperatures appeared more pronounced among older adults and insured patients. As the first systematic epidemiological study evaluating the relationship of environmental temperature with the risk of PH hospitalization, these findings provide important evidence to inform targeted climate health intervention strategies.

Previous studies have established robust associations between temperature and respiratory diseases (6, 27–29). For example, Chen et al. in a larger-scale analysis of 272 Chinese cities found that low temperatures posed higher respiratory mortality risk [RR: 1.53 (95% CI: 1.36–1.74)] than extremely high temperatures [RR: 1.36 (95% CI: 1.32–1.48)] (6). In Spain, Achebak et al. found a seasonal disparity, with hospitalizations peaking in cold seasons and mortality peaking during high temperatures (27). Gasparrini et al. through a multi-country collaboration, emphasized the substantial global mortality burden attribute to non-optimal temperatures (5). Extending this line of evidence, our study is the first to demonstrate that both extreme and moderate cold exposures are significantly related to elevated risk of PH hospitalization—a specific and severe cardiopulmonary condition not previously examined in this context. When comparing our findings to studies in different climatic zones, we observe both consistencies and variations. For instance, while our results align with research from other temperate cities in northern China (such as Gansu and Tianjin) regarding the shape of the temperature-mortality curve (30–32), they differ from findings in tropical climates or northeastern China where populations may have a higher heat tolerance (8, 33). This comparison underscores the need for region-specific public health policies.

While the primary aim of this study was to establish the epidemiological association, the observed links between temperature extremes and PH hospitalizations can be contextualized within existing physiological knowledge. Temperature fluctuations, particularly cold exposure, are known to trigger systemic vasoconstriction and increase peripheral vascular resistance (34). For patients with pre-existing pulmonary hypertension, whose right ventricular function is often already compromised, this increased afterload can precipitate acute cardiac strain or failure (35). Furthermore, extreme temperatures can induce systemic inflammatory responses and oxidative stress, potentially exacerbating endothelial dysfunction—a key feature of pulmonary vascular pathology (36). Although our study does not directly test these pathways, they offer a plausible biological framework for our findings and highlight important avenues for future experimental or clinical research.

Stratified analyses showed marked heterogeneity in temperature-related PH hospitalization risk across population subgroups, reflecting distinct vulnerability patterns (37). Women exhibited greater significantly to temperature-related PH hospitalization than men, potentially attributable to heightened thermoregulatory system sensitivity to environmental fluctuations in females (37, 38). Individuals aged < 65 years showed significantly higher hospitalization risk during extreme and moderate cold compared with older adults. This may be explained by greater outdoor exposure and relatively lower protective awareness among working-age populations (39). Studies have indicated that Shanghai’s rainy summer limits outdoor activity, while dry and stable winter conditions increase cold exposure among younger adults (40). Conversely, older adults (≥65 years) exhibited greater sensitivity to high temperatures, likely due to age-related deterioration of thermoregulatory function and higher prevalence of cardiovascular comorbidities (20). Notably, uninsured individuals showed a higher PH-related hospitalization risk in cold environments. Potential mechanisms include inadequate chronic disease management due to lack of regular care and delayed healthcare-seeking behavior driven by economic constrains, both of which may exacerbate health outcomes (41). Experimental evidence supports our findings: Sydykov et al. observed increased pulmonary artery pressure following cold exposure in patients, indicating an elevated pulmonary vascular response to cold (35). Animal experiments further demonstrated that chronic cold exposure exacerbates pulmonary hypertension in rat pulmonary arteries (34). Our findings align with research greater cold effects in southern cities and greater heat effects in northern cities. This geographical disparity may reflect adaptive responses to local climates: centralized heating systems in northern cities enhance cold adaptation, while widespread air conditioning use in southern cities like Shanghai mitigates summer heat exposure among residents (42).

Our study has several notable strengths. First, the use of time-series analysis allowed us to identify temporal trends, seasonality, and periodicity in PH hospitalizations, providing a comprehensive understanding of how temperature fluctuations impact hospitalization risk over time, including delayed effects. Second, the application of a distributed lag non-linear model enabled simultaneous characterization of non-linear exposure-response relationships and lag structures, improving the precision of effect estimation and offering nuanced insight into the timing of temperature-related risks. Third, subgroup analyses by demographic characteristics allowed identification of vulnerable groups, including females, younger adults, and uninsured individuals. Fourth, by extending the focus beyond common respiratory health outcomes like COPD and low temperature effects, our study addressed a critical research gap by systematically evaluating both extreme and moderate temperature exposures in relation to PH, a previously understudied condition. Collectively, these strengths enhance the existing literature and provide a robust evidence base for targeted interventions.

Our study also has some limitations. First, being a single-city study, to some extent, limits the generalizability of the findings to broader populations, especially those with different regional or socioeconomic characteristics. Caution should be exercised when extrapolating the results to other regions or countries. Second, relying the use of meteorological data from a single monitoring station may introduce exposure misclassification due to spatial variability in temperature across Shanghai, potentially biasing the results toward the null. Third, due to the lack of daily air pollution data (e.g., PM_2.5_, NO_2_, O_3_) for the study period, we were unable to adjust for these potential confounders in our models. As temperature and air pollution are often correlated, this may have resulted in uncontrolled confounding bias in the estimated associations. Future studies with access to comprehensive air quality monitoring data are needed to disentangle the independent effects of temperature from those of air pollution. Nevertheless, our adjustment for humidity and seasonal trends provides partial control for environmental factors, and sensitivity analyses suggested that our main findings were robust to alternative model specifications. Fourth, given the observational design, this study cannot establish causality between temperature exposure and PH hospitalizations, despite rigorous adjustment for confounders. Finally, given the relatively limited sample size in this study, we selected the 5th and 95th percentiles to ensure statistical stability. Future research with larger datasets or multi-center collaborations would be better positioned to examine associations at more extreme percentiles (e.g., 1st and 99th).

## Conclusion

This pioneering time-series analysis demonstrates that both extreme and moderate cold temperatures significantly increase the risk of PH hospitalization, with heightened vulnerability observed among females, individuals aged < 65 years, and those without medical insurance. These findings provide actionable evidence for developing targeted interventions to reduce the temperature-associated burden of PH hospitalizations, particularly among high-risk subgroups.

## Data Availability

The datasets used and/or analyzed during the current study are available from the corresponding author upon reasonable request.

## References

[1] Maron BA. Revised definition of pulmonary hypertension and approach to management: a clinical primer. *J Am Heart Assoc*. (2023) 12:e029024. 10.1161/JAHA.122.029024 37026538 PMC10227272

[2] Lai YC Potoka KC Champion HC Mora AL Gladwin MT. Pulmonary arterial hypertension: the clinical syndrome. *Circ Res*. (2014) 115:115–30. 10.1161/CIRCRESAHA.115.301146 24951762 PMC4096686

[3] Roy A Alam MA Kim Y Hashizume M. Association between daily ambient temperature and drug overdose in Tokyo: a time-series study. *Environ Health Prev Med*. (2022) 27:36. 10.1265/ehpm.21-00044 36171116 PMC9556974

[4] Cao R Wang Y Huang J He J Ponsawansong P Jin J et al. The mortality effect of apparent temperature: a multi-city Study in Asia. *Int J Environ Res Public Health*. (2021) 18:4675. 10.3390/ijerph18094675 33924779 PMC8124769

[5] Gasparrini A Guo Y Hashizume M Lavigne E Zanobetti A Schwartz J et al. Mortality risk attributable to high and low ambient temperature: a multicountry observational study. *Lancet*. (2015) 386:369–75. 10.1016/S0140-6736(14)62114-0 26003380 PMC4521077

[6] Chen R Yin P Wang L Liu C Niu Y Wang W et al. Association between ambient temperature and mortality risk and burden: time series study in 272 main Chinese cities. *BMJ*. (2018) 363:k4306. 10.1136/bmj.k4306 30381293 PMC6207921

[7] Jia H Xu J Ning L Feng T Cao P Gao S et al. Ambient air pollution, temperature and hospital admissions due to respiratory diseases in a cold, industrial city. *J Glob Health*. (2022) 12:04085. 10.7189/jogh.12.04085 36243957 PMC9569423

[8] Wu Y Liu X Gao L Sun X Hong Q Wang Q et al. Short-term exposure to extreme temperature and outpatient visits for respiratory diseases among children in the northern city of China: a time-series study. *BMC Public Health.* (2024) 24:341. 10.1186/s12889-024-17814-5 38302889 PMC10832290

[9] Zhao Q Zhao Y Li S Zhang Y Wang Q Zhang H et al. Impact of ambient temperature on clinical visits for cardio-respiratory diseases in rural villages in northwest China. *Sci Total Environ*. (2018) 612:379–85. 10.1016/j.scitotenv.2017.08.244 28858748

[10] Turner LR Barnett AG Connell D Tong S. Ambient temperature and cardiorespiratory morbidity: a systematic review and meta-analysis. *Epidemiology*. (2012) 23:594–606. 10.1097/EDE.0b013e3182572795 22531668

[11] Zhao Y Huang Z Wang S Hu J Xiao J Li X et al. Morbidity burden of respiratory diseases attributable to ambient temperature: a case study in a subtropical city in China. *Environ Health*. (2019) 18:89. 10.1186/s12940-019-0529-8 31651344 PMC6814053

[12] Danesh Yazdi M Wei Y Di Q Requia WJ Shi L Sabath MB et al. The effect of long-term exposure to air pollution and seasonal temperature on hospital admissions with cardiovascular and respiratory disease in the United States: a difference-in-differences analysis. *Sci Total Environ*. (2022) 843:156855. 10.1016/j.scitotenv.2022.156855 35750164 PMC10007814

[13] Achebak H Rey G Lloyd SJ Quijal-Zamorano M Méndez-Turrubiates RF Ballester J. Ambient temperature and risk of cardiovascular and respiratory adverse health outcomes: a Nationwide cross-sectional study from Spain. *Eur J Prev Cardiol*. (2024) 31:1080–9. 10.1093/eurjpc/zwae021 38364198

[14] Sun S Laden F Hart JE Qiu H Wang Y Wong CM et al. Seasonal temperature variability and emergency hospital admissions for respiratory diseases: a population-based cohort study. *Thorax*. (2018) 73:951–8. 10.1136/thoraxjnl-2017-211333 29622691

[15] Zhao Q Guo Y Ye T Gasparrini A Tong S Overcenco A et al. Global, regional, and national burden of mortality associated with non-optimal ambient temperatures from 2000 to 2019: a three-stage modelling study. *Lancet Planet Health*. (2021) 5:e415–25. 10.1016/S2542-5196(21)00081-4 34245712

[16] Brauer M Roth GA Aravkin AY Zheng P Abate KH Abate YH et al. Global burden and strength of evidence for 88 risk factors in 204 countries and 811 subnational locations, 1990–2021: a systematic analysis for the Global Burden of Disease Study 2021. *Lancet.* (2024) 403:2162–203. 10.1016/S0140-6736(24)00933-4 38762324 PMC11120204

[17] Antimicrobial Resistance Collaborators. Global burden of bacterial antimicrobial resistance in 2019: a systematic analysis. *Lancet.* (2022) 399:629–55. 10.1016/S0140-6736(21)02724-0 35065702 PMC8841637

[18] Chen R Li T Cai J Yan M Zhao Z Kan H. Extreme temperatures and out-of-hospital coronary deaths in six large Chinese cities. *J Epidemiol Community Health*. (2014) 68:1119–24. 10.1136/jech-2014-204012 25108018

[19] Yang C Meng X Chen R Cai J Zhao Z Wan Y et al. Long-term variations in the association between ambient temperature and daily cardiovascular mortality in Shanghai, China. *Sci Total Environ*. (2015) 538:524–30. 10.1016/j.scitotenv.2015.08.097 26318688

[20] Ma W Chen R Kan H. Temperature-related mortality in 17 large Chinese cities: How heat and cold affect mortality in China. *Environ Res*. (2014) 134:127–33. 10.1016/j.envres.2014.07.007 25127523

[21] Liu M Zhu X Pan C Chen L Zhang H Jia W et al. Spatial variation of near-surface CO2 concentration during spring in Shanghai. *Atmospheric Pollut. Res.* (2016) 7:31–9. 10.1016/j.apr.2015.07.002

[22] Qing M Guo Y Yao Y Zhou C Wang D Qiu W et al. Effects of apparent temperature on daily outpatient and inpatient visits for cause-specific respiratory diseases in Ganzhou, China: a time series study. *Environ Health Prev Med*. (2024) 29:20–20. 10.1265/ehpm.23-00188 38522902 PMC10965414

[23] Duan J Wang X Zhao D Wang S Bai L Cheng Q et al. Risk effects of high and low relative humidity on allergic rhinitis: time series study. *Environ Res*. (2019) 173:373–8. 10.1016/j.envres.2019.03.040 30954910

[24] Terada S Nishimura H Miyasaka N Fujiwara T. Ambient temperature and preterm birth: a case-crossover study. *BJOG*. (2024) 131:632–40. 10.1111/1471-0528.17720 37984435

[25] Zhang R Peng Z Meng Y Song H Wang S Bi P et al. Temperature and influenza transmission: risk assessment and attributable burden estimation among 30 cities in China. *Environ Res*. (2022) 215(Pt 1):114343. 10.1016/j.envres.2022.114343 36115415

[26] Li H Wu J Wang A Li X Chen S Wang T et al. Effects of ambient carbon monoxide on daily hospitalizations for cardiovascular disease: a time-stratified case-crossover study of 460,938 cases in Beijing, China from 2013 to 2017. *Environ Health*. (2018) 17:82. 10.1186/s12940-018-0429-3 30477579 PMC6258455

[27] Achebak H Garcia-Aymerich J Rey G Chen Z Méndez-Turrubiates RF Ballester J. Ambient temperature and seasonal variation in inpatient mortality from respiratory diseases: a retrospective observational study. *Lancet Reg Health Eur*. (2023) 35:100757. 10.1016/j.lanepe.2023.100757 38115961 PMC10730325

[28] Lin Z Gu Y Liu C Song Y Bai C Chen R et al. Effects of ambient temperature on lung function in patients with chronic obstructive pulmonary disease: a time-series panel study. *Sci Total Environ*. (2018) 61:360–5. 10.1016/j.scitotenv.2017.11.035 29156256

[29] Bunker A Wildenhain J Vandenbergh A Henschke N Rocklöv J Hajat S et al. Effects of air temperature on climate-sensitive mortality and morbidity outcomes in the elderly; a systematic review and meta-analysis of epidemiological evidence. *EBioMedicine*. (2016) 6:258–68. 10.1016/j.ebiom.2016.02.034 27211569 PMC4856745

[30] Li D Dong J Liu X Ge J Shu J Zhu L et al. Exploring the impact of three meteorological factors and their specific effect sizes on chronic obstructive pulmonary disease admission in Qingyang, China. *Sci Rep*. (2025) 15:24803. 10.1038/s41598-025-10207-7 40640439 PMC12246443

[31] Zhang X Xu K Li Q Zhu A Yu J Liu M et al. Exploring the impact of ambient temperature on respiratory diseases admissions, length of Stay, and hospitalization costs in Lanzhou City, based on distributed lag non-linear model. *Climate Serv.* (2024) 34:100481. 10.1016/j.cliser.2024.100481

[32] Chang H Li M Wang Y Cui L Li T. Acute effects of low temperatures and cold waves on elderly infectious pneumonia mortality - Jinan City, Shandong Province, China, 2014-2022. *China CDC Wkly*. (2024) 6:77–82. 10.46234/ccdcw2024.017 38410531 PMC10894711

[33] Ma Y Zhang Y Jiao H Cheng B Li H An X et al. Extreme temperatures and respiratory mortality in the capital cities at high latitudes in Northeast China. *Urban Climate.* (2022) 44:101206. 10.1016/j.uclim.2022.101206

[34] Ishii T Shimo Y. Cooling-induced supersensitivity to acetylcholine in the isolated airway smooth muscle of the rat. *Naunyn Schmiedebergs Arch Pharmacol*. (1985) 329:167–75. 10.1007/BF00501208 3847775

[35] Sánchez-Gloria JL Carbó R Buelna-Chontal M Osorio-Alonso H Henández-Díazcouder A de la Fuente-León RL et al. Cold exposure aggravates pulmonary arterial hypertension through increased miR-146a-5p, miR-155-5p and cytokines TNF-α, IL-1β, and IL-6. *Life Sci*. (2021) 287:120091. 10.1016/j.lfs.2021.120091 34717910

[36] Crosswhite P Sun Z. Inhibition of phosphodiesterase-1 attenuates cold-induced pulmonary hypertension. *Hypertension*. (2013) 61:585–92. 10.1161/HYPERTENSIONAHA.111.00676 23319544 PMC4050371

[37] Greenfield AM Alba BK Giersch GEW Seeley AD. Sex differences in thermal sensitivity and perception: implications for behavioral and autonomic thermoregulation. *Physiol Behav*. (2023) 263:114126. 10.1016/j.physbeh.2023.114126 36787810

[38] Lautenbacher S Strian F. Sex differences in pain and thermal sensitivity: the role of body size. *Percept Psychophys*. (1991) 50:179–83. 10.3758/bf03212218 1945739

[39] Glanz K Buller DB Saraiya M. Reducing ultraviolet radiation exposure among outdoor workers: state of the evidence and recommendations. *Environ Health*. (2007) 6:22. 10.1186/1476-069X-6-22 17686155 PMC1995198

[40] Ma W Xu X Peng L Kan H. Impact of extreme temperature on hospital admission in Shanghai, China. *Sci Total Environ*. (2011) 409:3634–7. 10.1016/j.scitotenv.2011.06.042 21752430

[41] Li X Zhang W. The impacts of health insurance on health care utilization among the older people in China. *Soc Sci Med*. (2013) 85:59–65. 10.1016/j.socscimed.2013.02.037 23540367

[42] Sun C Li A Gui R Xue Y Cao Y Chen G. The impact of cold spells and heat waves frequencies on the prevalence and incidence of stroke in middle-to-elderly age population in China: evidence from the China Health and Retirement Longitudinal Study (CHARLS). *Int J Biometeorol*. (2025) 69:1153–65. 10.1007/s00484-025-02885-9 40072559

