# Supplementary material for: Extreme and moderate temperatures and risk of hospitalizations for pulmonary hypertension: an 11-year time-series study in Shanghai, China
Source: Front Med (Lausanne). 2026 Mar 23;13:1771445. doi: 10.3389/fmed.2026.1771445 (PMC13050875; doi:10.3389/fmed.2026.1771445)
Supplement: Supplementary file 1 [file Table_1.docx]

**Supplementary Materials**

**Title:** Extreme and moderate temperatures and risk of hospitalizations for pulmonary hypertension: An eleven-year time-series study in Shanghai, China

**Figure legends**

**Fig. S1.** A time-series diagram of daily average temperature and the PH hospitalized cases over the duration of study. Abbreviations as in Table 1

**Fig. S2**. Spearman correlation between meteorological conditions during the period 2013-2023.

**Fig. S3**. The effect of daily average temperature on risk of PH hospitalizations after adjusting the degrees of freedom (df = 1,2,4,5,6,7) of relative humidity.

**Fig. S4**. The effect of daily average temperature on risk of PH hospitalizations after adjusting the degrees of freedom (df = 8, 9, 10) of time trend variable.

**Fig. S5**. Exposure-response relationship between daily mean temperature and the risk of PH hospitalization, with the minimum-risk temperature as the reference. Abbreviations are shown in Table 1.
