# Supplementary figures and images for: Extreme and moderate temperatures and risk of hospitalizations for pulmonary hypertension: an 11-year time-series study in Shanghai, China
Source: Front Med (Lausanne). 2026 Mar 23;13:1771445. doi: 10.3389/fmed.2026.1771445 (PMC13050875; doi:10.3389/fmed.2026.1771445)

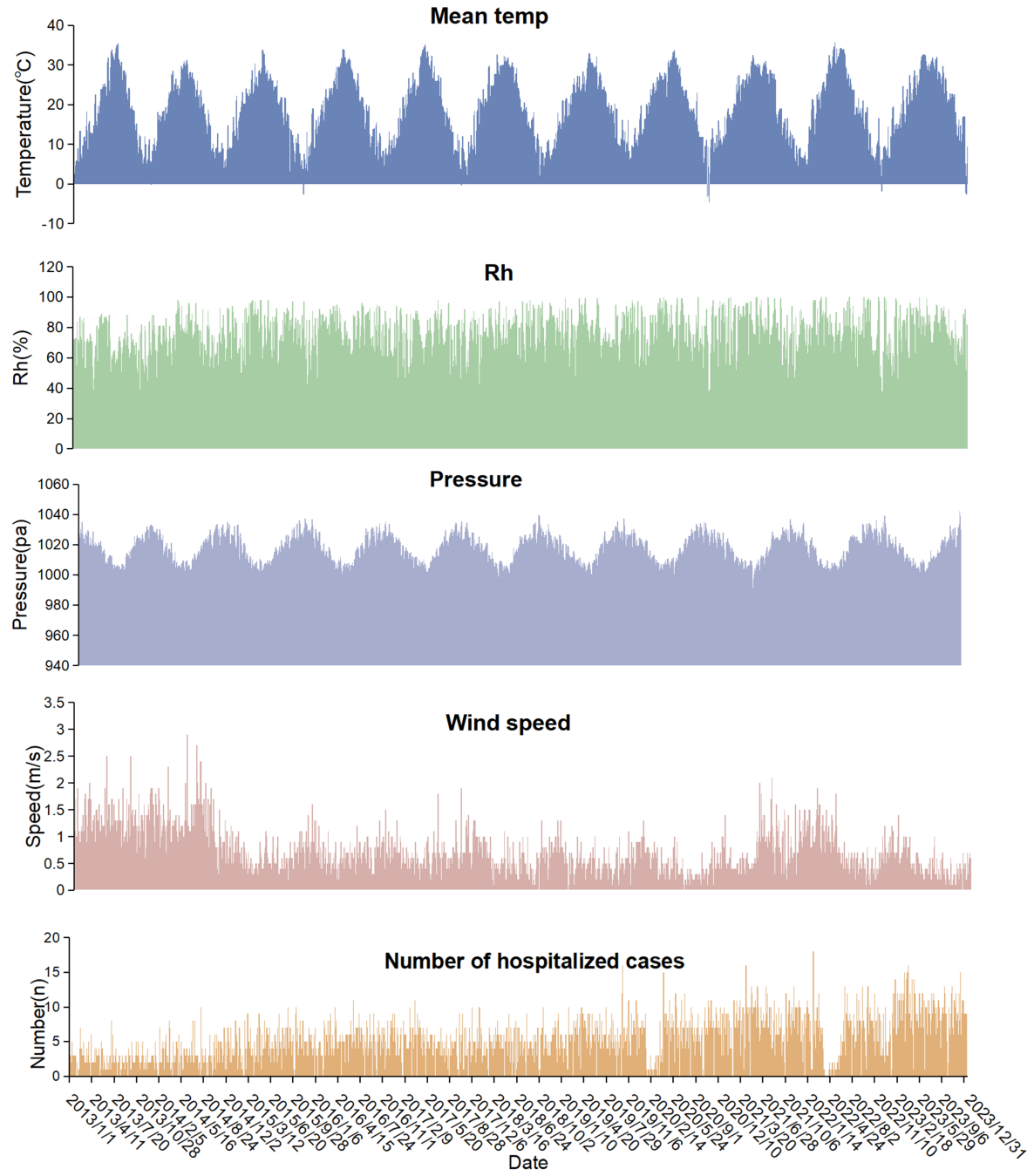

Supplement: Supplementary file 2 [file Image_1.tif]

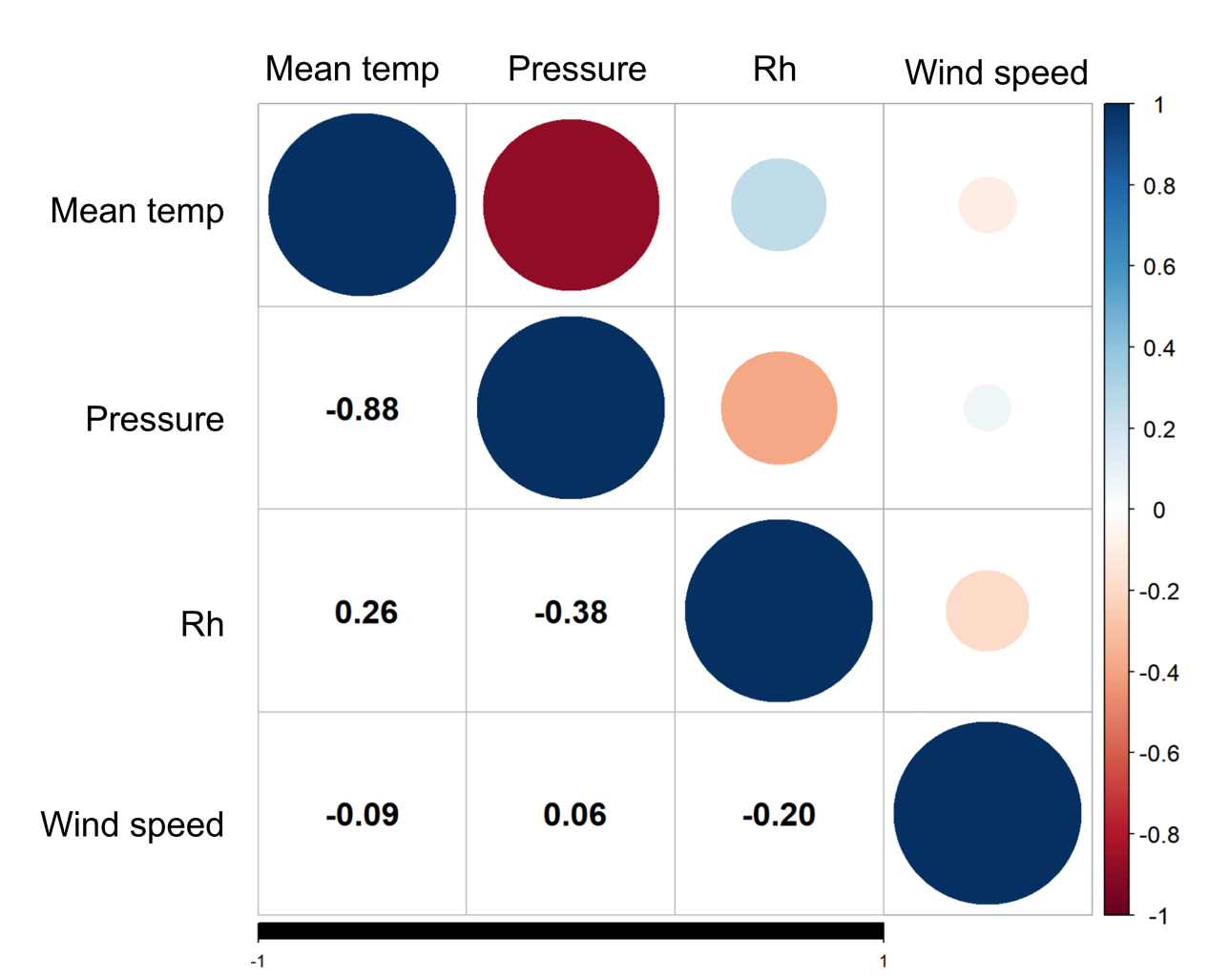

Supplement: Supplementary file 3 [file Image_2.tif]

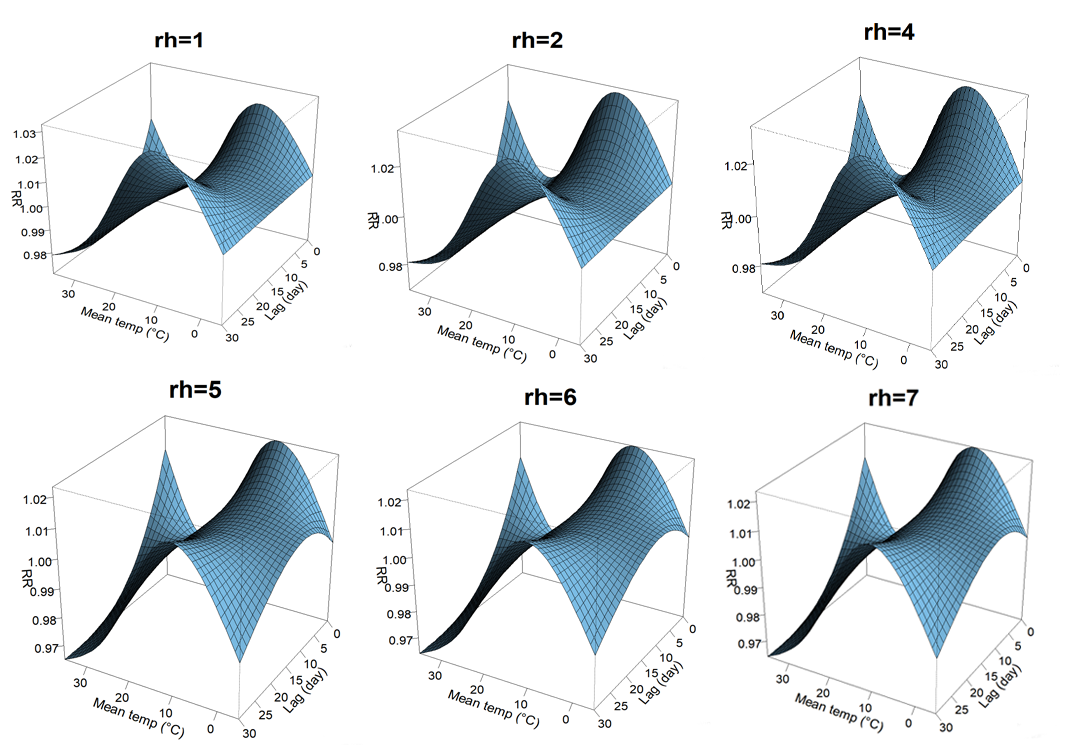

Supplement: Supplementary file 4 [file Image_3.tif]

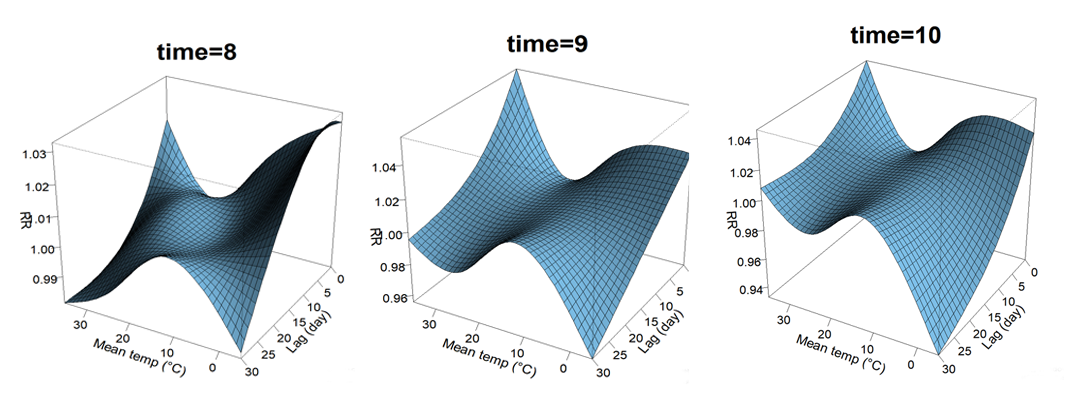

Supplement: Supplementary file 5 [file Image_4.tif]

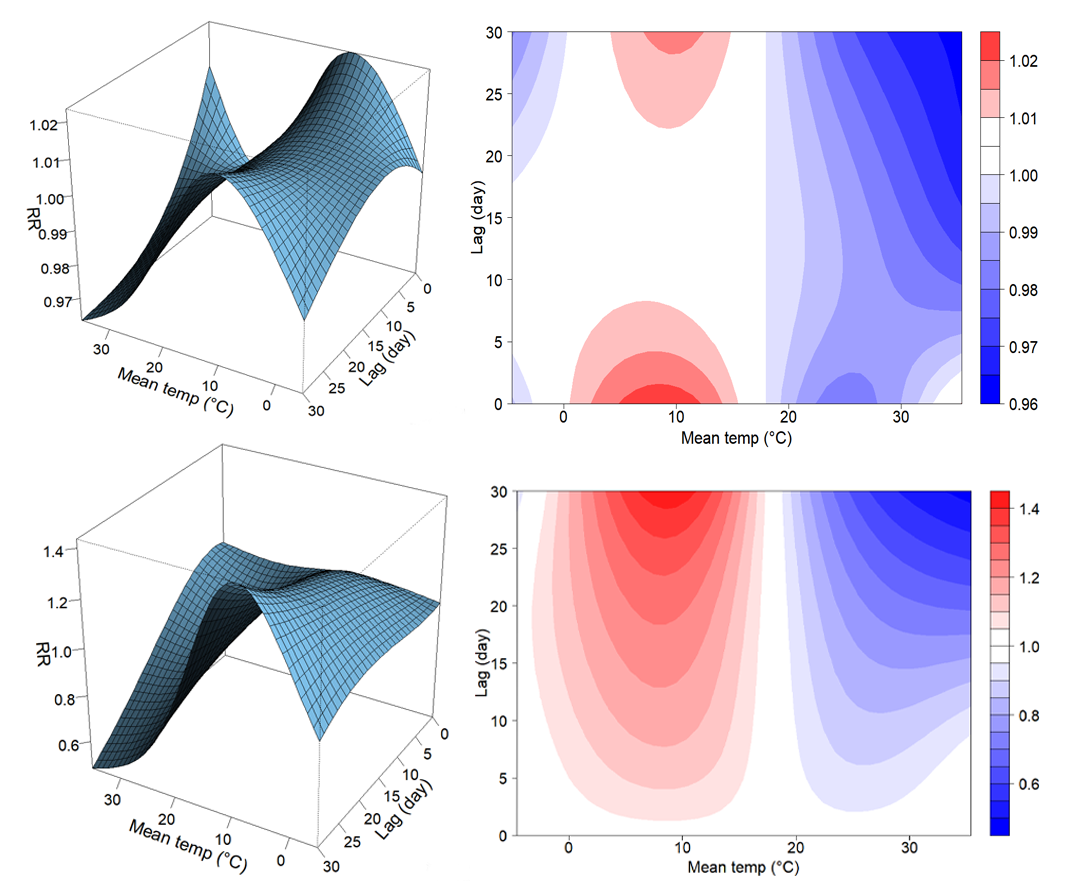

Supplement: Supplementary file 6 [file Image_5.tif]
